# Potential Use of Essential Oil Isolated from* Cleistocalyx operculatus* Leaves as a Topical Dermatological Agent for Treatment of Burn Wound

**DOI:** 10.1155/2018/2730169

**Published:** 2018-03-05

**Authors:** Gia-Buu Tran, Nghia-Thu Tram Le, Sao-Mai Dam

**Affiliations:** Institute of Biotechnology and Food Technology, Industrial University of Ho Chi Minh City, 12 Nguyen Van Bao Street, Go Vap District, Ho Chi Minh City, Vietnam

## Abstract

Several herbal remedies have been used as topical agents to cure burn wound, one of the most common injuries in worldwide. In this study, we investigated the potential use of* Cleistocalyx operculatus* essential oil to treat the burn wound. We identified a total of 13 bioactive compounds of essential oil, several of which exhibited the anti-inflammatory and antimicrobial activities. Furthermore, the essential oil showed the antibacterial effect against* S. aureus* but not with* P. aeruginosa.* The supportive effect of essential oil on burn wound healing process also has been proven. Among three groups of mice, wound contraction rate of essential oil treated group (100%) was significantly higher than tamanu oil treated (79%) and control mice (71%) after 20 days (0.22 ± 0.03 versus 0.31 ± 0.02 cm^2^, resp., *p* < 0.05). Histological studies revealed that burn wounds treated with essential oil formed a complete epidermal structure, thick and neatly arranged fibers, and scattered immune cells in burn wound. On the contrary, saline treated burn wound formed uneven epidermal layer with necrotic ulcer, infiltration of immune cells, and existence of granulation tissue. This finding demonstrated* Cleistocalyx operculatus* essential oil as promising topical dermatological agent to treat burn wound.

## 1. Introduction

Burn, one of the most common household injuries, is defined as a type of damage of skin or other tissues caused by exposure to heat, chemicals, electric currents, flame, hot liquid, hot metal or object, steam, radiation from X-ray, sunlight, ultraviolet, and so forth. Among them, the thermal injuries are leading cause of burn (86%), followed by electrical sources (4%) and contacting with chemical sources (3%), and other sources of burns (7%) [1]. Note that flame and scald burns account for the majority of burns in children and adults. Thermal burn causes not only a small or local injury which can be treated at home or outpatient but also the severe and fatal injuries which require the in-hospital treatment. The World Health Organization estimates that thermal burns account for approximated 6.6 million injuries and 300 thousand deaths annually over the world [1]. Furthermore, Kemp et al. (2017) also suggest that 25,000 children who suffered from burns or scald require the Emergency Department admission in English and Wales each year, of which 3,800 patients must receive in-hospital treatment [2]. The severity of burn is evaluated by the extent and the depth of burn. The extent of burn is estimated through total body surface area burn (% TBSA) whereas the depth of burn is estimated by the deep extent of injury into the epidermis or dermis. If the burn extent involves only the epidermis, thus it is classified as superficial burn (first degree). When the burn involves epidermis and dermis, it is categorized as partial thickness burn (second degree). The other burn is full-thickness burn (third degree) which extend into subcutaneous fat or deeper. Thermal burns resulted in both local injuries and a systemic response, in case of severe burn (% TBSA > 20%). Deep and widespread burns may cause many complications such as infection, hypovolemia, hypothermia, blood clotting, scarring, joint mobility problem, and posttraumatic stress disorder. In superficial and partial thickness of burns, topical antibacterial regimes with antibiotic ointments or cream and/or absorbent dressing to cover the burn wound are recommended. Otherwise, in deep partial and full thickness of burns, the surgical excision of damaged tissue and skin grafting are performed to cure the wound.

Several medical plants exerting antibacterial activity and/or wound healing potential have been applied for treating burn injuries such as* Aloe vera*,* Achillea millefolium*,* Carica papaya*, and* Datura alba* [10]. Herbal preparations may be obtained from a variety of parts of plants (fruit, leaf, bulb, stem, root, pollen, whole plant, and seed) and extraction forms (oil, acetone, methanol, ethanol, hydroalcoholic, and aqueous extract). In Polynesia and Southeast Asia, tamanu oil which is produced from* Calophyllum inophyllum *is the well-known and common use product to heal thermal burn wound. Tamanu oil has been proven as promising topical remedy which exerts acceleration of skin healing process, antineuralgic, antioxidant, anti-inflammatory, and antimicrobial effects [11]. Note that the number of researches using vegetable oil and essential oil to treat burn wound has been increased. Dursun et al. (2003) suggested that thymus essential oil could reduce NO level induced by burn and enhance the formation of new tissue in burn wound [12]. The beneficial effect of* Nigella sativa *seed oil on burn wound healing process also was proven [13]. Furthermore, Khedir et al. (2016) observed that* Pistacia lentiscus *fruit oil accelerated wound contraction in CO_2_ laser burned wound model [14]. These findings consolidate the idea using the vegetable and vegetable oils for treating burn wound.

*Cleistocalyx operculatus *(Roxb.) Merr & Perry is a well-known medicinal plant in Asia. It is grown and widely spread in Vietnam, China, Malaysia, Myanmar, Thailand, Sri Lanka, India, Nepal, and other tropical countries. Leaves and buds are two parts of* C. operculatus* commonly used for treating gastrointestinal disorder and dermatophytic infection for many years [15]. Furthermore, the other beneficial effects of* C. operculatus *such as anticancer, antihyperglycemic hypolipidemic, and cardiotonic effects are well documented [16, 17]. In addition, Dũng et al. (1994) have analyzed the chemical composition of essential oil isolated from* Cleistocalyx operculatus* leaves [18]. Recently, Dosoky et al. (2015) proved that essential oil of* Cleistocalyx operculatus* leaves possessed a strong antimicrobial effect and cytotoxicity to cancer cell lines [19]. However, application of essential oil isolated from* Cleistocalyx operculatus* leaves on wound healing, especially in thermal injury, has not been elucidated yet. Therefore, we investigated the effect of essential oil isolated from* Cleistocalyx operculatus* leaves on burn wound model in this study.

## 2. Materials and Methods

### 2.1. Collection and Preparation of* Cleistocalyx operculatus* Essential Oil

*Cleistocalyx operculatus* leaves were purchased from local herbal supplier in Go Vap District, Ho Chi Minh City, Vietnam (Thanh Binh Medicinal Plants and Herbals Co., Vietnam). Air-dried and ground leaves were subjected to hydrodistillation for 4 hours at 100°C in 15% NaCl solution using a Clevenger apparatus [20]. The essential oils were collected over aqueous phase amd transferred into 1.5 mL tube, after which essential oil was stored in dark chamber at 4°C prior to GC/MS analysis and bioactivities testing.

### 2.2. Gas Chromatography-Mass Spectrometry (GC-MS) Analysis

The GC-MS analysis of the essential oil was performed at Department of Analytical Chemistry, University of Science, Vietnam National University of Ho Chi Minh City with the given protocol. Briefly, chemical compositions of essential oil were analyzed on a GC-MS Aligent 6890 system equipped with a splitless mode injector and DB–5MS column (30 m × 0.25 mm ID, film thickness 0.25 *μ*m from Aligent Technologies, USA). The GC injector temperature was set at 250°C. A 1 mL volume of 2,000 ppm oil solution (1 hot water: 10 methanol) was injected. Helium in constant pressure was used as carrier gas at flow rate of 1.0 mL/min. The oven initial temperature was maintained 60°C for 1 min and heated at 10°C/min until oven temperature reached 200°C, and the oven was kept in this temperature for 5 min. Then oven was heated at 20°C/min to 280°C and then kept for 1 min. The temperature of transfer line was set at 280°C. For GC-MS analysis, an electron ionization with ionization energy of 1700 eV was used, covering a mass range from 40 to 450 mz. The compounds were identified by NIST MS Search version 2.0.

### 2.3. Antimicrobial Activity

The antimicrobial activity of essential oil was determined by the agar diffusion method. The following bacterial strains* Staphylococcus aureus* ATCC 6538 and* Pseudomonas aeruginosa* ATCC 9027, which are considered as two common opportunistic bacteria in skin and mucous membrane [21], were employed for screening the antimicrobial activity of* C. operculatus *essential oil. Briefly, the tested microorganisms (0.1 ml of 1 × 10^8^ CFU/ml) were inoculated on LB agar. Then the sterilized filter paper discs (6 mm in diameter) were impregnated with 20 *μ*L of* Cleistocalyx operculatus* essential oil or tamanu oil. The discs were placed in LB agar plates, after which the plates were incubated at 37°C for 24 hours. The commercial antibiotic discs (Gentamycin, Nam Khoa Biotek Co.) and tamanu oil (Inopilo, Binh Minh Pharmaceutical Joint Co. Ltd.) were used as positive controls. The diameters of inhibition zones were measured in millimeters.

### 2.4. Establishment of Burned Mouse Model

Eight-week-old male Swiss albino mice were obtained from Pasteur Institute of Ho Chi Minh City, weighing approximately 30–32 g. The animals were randomly divided into polycarbonate cages with 4 mice for each cage. They were housed under standard husbandry conditions with 12 h light-dark cycle (8:00–20:00) for at least 1 week to acclimate with laboratory environment. They were supplied ad libitum with standard chow and distilled water. The experimental procedure was strictly in compliance with the Declaration of Helsinki (1964). Briefly, twelve healthy mice were randomly divided into 3 groups with 4 mice per group and treated as the protocol of Tavares Pereira et al. (2012) with some modifications [22]. Mice were anesthetized with diethyl ether for 3 min, then the hair on back of mice was removed using razor. The dorsal proximal region was antisepsis with polyvinyl pyrrolidone iodine. Thermal lesion was generated by a massive aluminum bar 10 mm in diameter preheated to 100 ± 5°C/10 min. The probe was kept to contact with mouse skin for 15 sec. After that, the burn wound was treated with an indicated volume of saline, tamanu oil, or diluted* C. operculatus* essential oil (50 *μ*l/lesion) once per day for 20 days. Tamanu oil (Inopilo, Binh Minh Pharmaceutical Joint Co. Ltd.) was used as reference treatment. Diluted* C. operculatus* essential oil (1% solution) was prepared by dissolving the essential oil in 0.1% DMSO and Tween 20 solution. The burned area of mice was measured after 10 days and 20 days and the results were presented as square centimeter (cm^2^).

### 2.5. Histological Study

At the end of experiment (20 days), all mice were anesthetized with diethyl ether and then euthanized by carbon dioxide. The skin of burned area was collected and preserved in 10% formalin. The sample was processed for histological studies with Hematoxylin and Eosin staining in Division of Pathological Anatomy, the Cancer Diagnosis and Treatment Centre of Military Hospital 175 with given protocol [23].

### 2.6. Statistical Analysis

All experiments were repeated in triplicate. Statistical analysis was performed using Statgraphics Centurion XVI software (Statpoint Technologies Inc., Warrenton, Virginia, USA). The data were presented as mean ± standard deviation. Differences between means of different groups were analyzed using ANOVA variance analysis followed with multiple range tests, and the criterion of statistical significance was set as *p* < 0.05.

## 3. Results and Discussions

### 3.1. Screening Bioactive Compounds of* C. operculatus* Essential Oil

**Figure 1:**
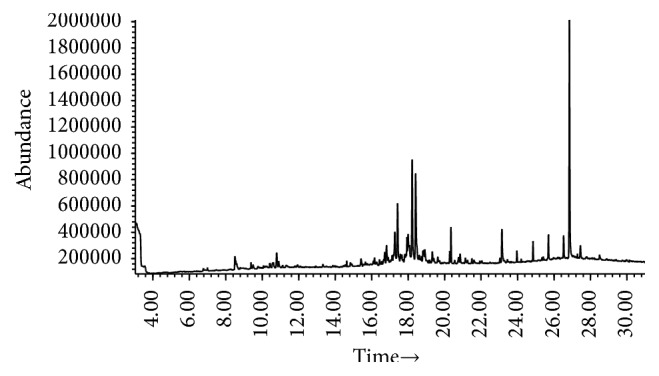
*GC/MS chromatogram of essential oil extracted from Cleistocalyx operculatus leaves*. A total of 13 compounds were identified in CLO: 6-camphenol; isopinocarveol; p-cymen-8-ol; (−)-myrtenol; I-verbenone; cis-carveol; ethaneperoxoic acid, 1-cyano-4,4-dimethyl-1-phenylpentyl ethaneperoxate; (+)-carotol; caryophyllene oxide; (−)-globulol; 2-(4a,8-dimethyl-2,3,4,4a,5,6-hexahydronaphthalen-2-yl)propan-1-; and longipinocarvone. Among them, many bioactive compounds identified in CLO exhibited antimicrobial and/or anti-inflammatory activities. This finding supports the idea of using* Cleistocalyx operculatus *essential oil as topical agent for treatment of burn wound, at least to prevent the infection and sepsis.

*C. operculatus* leaves essential oil (CLO) has yellowish color and fragrant odor, with 0.1% yield. The presence of some active compounds in CLO was determined by GC-MS analysis, and data were recorded in Figure 1 and Table 1. Briefly, a total of 13 compounds were identified in CLO: 6-camphenol; isopinocarveol; p-cymen-8-ol; (−)-myrtenol; I-verbenone; cis-carveol; ethaneperoxoic acid, 1-cyano-4,4-dimethyl-1-phenylpentyl ethaneperoxate; (+)-carotol; caryophyllene oxide; (−)-globulol; 2-(4a,8-dimethyl-2,3,4,4a,5,6-hexahydronaphthalen-2-yl) propan-1-; and longipinocarvone. Most of bioactive compounds identified in CLO exhibited antimicrobial and/or anti-inflammatory activities, such as isopinocarveol, (−)-myrtenol; I- verbenone; cis-carveol; (+)-carotol; caryophyllene oxide; (−)-globulol. This finding indicated that CLO may be used as topical treatment, at least as the anti-infective and antiseptic agent, for burn wound. Therefore, the next question has been raised whether CLO could inhibit the growth and/or eliminate some common bacteria inhabited on burn wound or not.

### 3.2. Screening of Antibacterial Activity of* C. operculatus* Essential Oil

**Figure 2:**
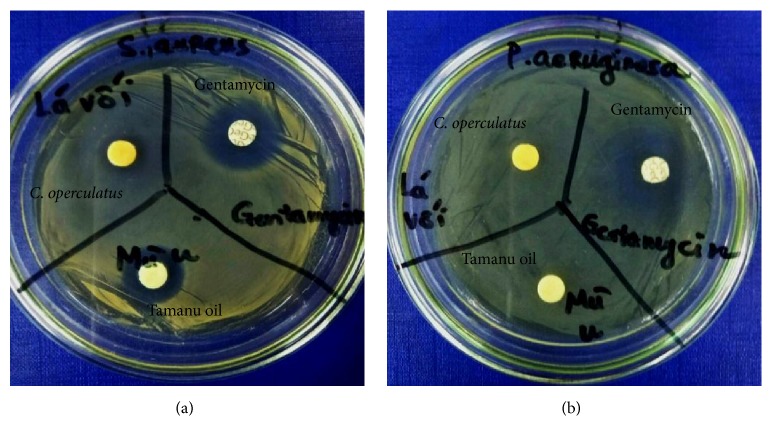
*Antimicrobial activity of C. operculatus essential oil*. Both CLO and commercial tamanu oil exhibited antibacterial activity against* S. aureus* whereas they did not inhibit the growth of* P. aeruginosa*. The diameter of inhibition zones against* S. aureus* of gentamicin (positive control) was highest (11.37 ± 0.15 mm), followed by tamanu oil (9.03 ± 0.31 mm) and CLO (7.17 ± 0.12 mm). On the contrary, only gentamicin showed the antibacterial activity against* P. aeruginosa (*12.07 ± 0.15 mm); both tamanu oil and CLO did not affect the growth of* P. aeruginosa. *This finding implies the potential use of CLO as anti-infective agent for burn wound treatment. The experiments were triplicated, and results were presented as mean ± standard deviation.

In previous report, Livimbi and Komolafe (2007) suggested that* S. aureus* was the most common bacteria isolated from burn wound, followed by* P. mirabilis, Streptococci *spp.,* P. aeruginosa, E. coli, Salmonella*, and* Klebsiella *spp. [21]. Therefore, we investigated antibacterial activity of* C. operculatus *essential oil against two bacteria commonly found on burn wound such as* S. aureus* and* P. aeruginosa* to prove the anti-infective efficiency of* C. operculatus* essential oil (CLO). Furthermore,* S. aureus* and* P. aeruginosa* also represent two types of bacteria, Gram positive and Gram negative bacteria, respectively. We found that both CLO and commercial tamanu oil exhibited antibacterial activity against* S. aureus* whereas they did not exhibit antibacterial activity against* P. aeruginosa* (Figure 2). The diameter of inhibition zones of gentamicin (positive control) against* S. aureus* was highest (11.37 ± 0.15 mm), followed by tamanu oil (9.03 ± 0.31 mm) and CLO (7.17 ± 0.12 mm, *p* < 0.05). On the contrary, only gentamicin showed the antibacterial activity against* P. aeruginosa (*12.07 ± 0.15), but* P. aeruginosa *was resistant with commercial tamanu oil and CLO (diameters of inhibition zones = diameter of dishes, 6 mm). These results were identical with previous reports [19, 24, 25]. In previous study, Nguyen et al. (2017) suggested that methanol extract of* C. operculatus* leaves could inhibit* S. aureus* but not hinder* P. aeruginosa* growth [24]. Furthermore, the antimicrobial activity of essential oil of* C. operculatus* leaves from Nepal against* S. aureus* has also been demonstrated [19]. However, Dung et al. (2008) indicated that essential oil of* C. operculatus* isolated from flower buds could inhibit both* S. aureus* and* P. aeruginosa* [16]. It may be explained that different parts of* C. operculatus* possess a variety of bioactive compounds which account for different antimicrobial activities of essential oils isolated from different parts of* C. operculatus.* In addition, although tamanu oil is effective remedy for burn wound treatment,* P. aeruginosa* is also resistant with commercial tamanu oil [25]. From these results, we suggested that CLO has antimicrobial activity against* S. aureus*, the most common skin wound opportunistic bacterium, but the effectiveness of CLO is lower than commercial tamanu oil. This finding implies the potential use of CLO as anti-infective agent for burn wound treatment. Next we investigated the wound contractive ability of* C. operculatus *essential oil on second-degree burn wound model.

### 3.3. Establishment of Second-Degree Burn Wound Model

**Figure 3:**
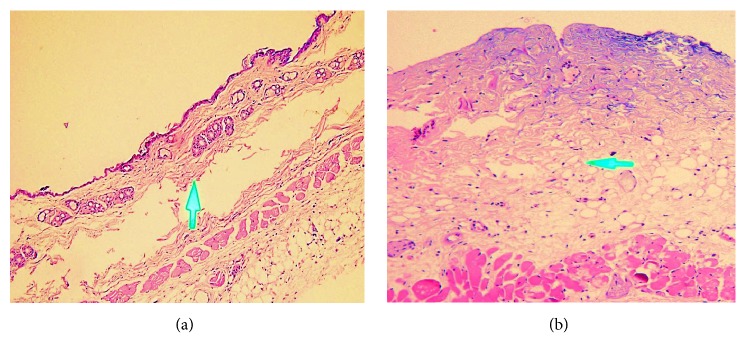
*Establishment of second-degree burn wound models*. Histological analysis showed that injury of burn wound was extended into both epidermis and dermis. In normal skin section, thick squamous epithelium covered the epidermis, and both of epidermis and dermis had the normal structure with several hair follicles and sebaceous glands (a). In burn wound skin section, epidermis and dermis lost their normal structure. Of note, squamous epithelium layer was removed, and epidermis was necrotized. Moreover, the underlying stromal tissue was swollen and congestive (b). These results indicated that the second-degree burn model was successfully established in experimental mice. The arrows indicated the border between the dermis and hypodermis.

The second-deep-degree burn is characterized by the extent of injury through the epidermis and into the dermis with painful, red, blistered, moist wound [1]. To confirm severity of burn wound, the histological examination of burned skin collected from burn lesion was performed with pathological experts from Division of Pathological Anatomy, the Cancer Diagnosis and Treatment Centre of Military Hospital 175. Histological analysis showed that injury of burn wound was extended into both epidermis and dermis. In normal skin section, thick squamous epithelium covered the epidermis, and both of epidermis and dermis had the normal structure with several hair follicles and sebaceous glands. In burn wound skin section, epidermis and dermis lost their normal structure. Of note, squamous epithelium layer was removed, and epidermis was necrotized. Moreover, the underlying stromal tissue was swollen and congestive. These results indicated that the second-degree burn model was successfully established in experimental mice (Figure 3).

### 3.4. Supportive Effect of* C. operculatus* Essential Oil on Burn Wound Model

**Figure 4:**
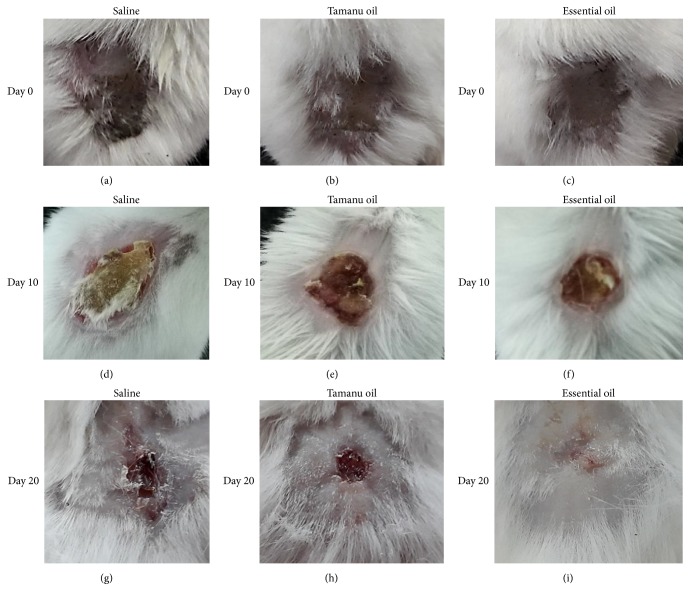
*Evaluation of the healing rate of burn wound in experimental mice*. At the beginning of experiment, all burn wounds were of similar sizes in three groups (a, b, c). In day 10, burn wound areas of tamanu oil (e) and essential oil treated mice (f) were smaller than saline treated group (d), and the significant difference of wound contraction between tamanu oil and essential oil treated mice was observed (*p* < 0.05). Furthermore, we found that burn wounds of essential oil treated mice were fully recovered (i). Burn wound contraction rate of tamanu oil treated mice (h) was significantly higher than that of saline treated groups (g) after 20 days. These results implied that* C. operculatus *essential oil has supportive effect on wound healing process and its efficiency was higher than the commercial tamanu oil.

We found that both tamanu oil and essential oil accelerated the wound contraction rate of burn wound after 10 days and 20 days (*p* < 0.05). At the beginning of the experiment, all burn wounds were of similar sizes in three groups. In day 10, burn wound areas of tamanu oil and CLO treated mice (0.57 ± 0.04 and 0.43 ± 0.03 mm^2^, accordingly) were smaller than saline treated group (0.73 ± 0.04 cm^2^), and the significant difference of wound contraction between tamanu oil and essential oil treated mice was observed (*p* < 0.05). Furthermore, essential oil group was fully recovered whereas the burn areas of tamanu oil treated and control groups remained after 20 days. Of note, wound healing process of tamanu oil treated mice was also significantly higher than control mice at this time-point (0.22 ± 0.03 versus 0.31 ± 0.02 cm^2^, respectively, *p* < 0.05). These results implied that* C. operculatus *essential oil has supportive effect on wound healing process and its efficiency was higher than the commercial tamanu oil (Table 2, Figure 4).

**Figure 5:**
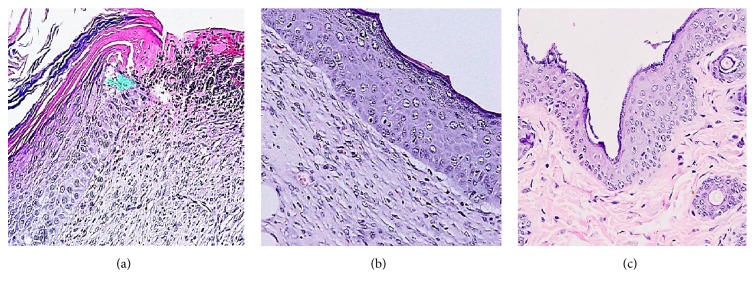
*Microscopic changes in burn wounds of experimental mice*. Histology studies revealed that burn wounds treated with essential oil developed complete epidermal structure (c). In tamanu oil treated mice, wounds were partially recovered with existence of coagulative necrosis region on epidermis and vascular congestion, swollen stromal tissue, and no matured hair follicles (b). On the contrary, saline treated mice formed uneven epidermal layer with the ulcer on epidermis layer and infiltration of immune cells (a). The highlighted part in (a) pointed out the ulcer region with infiltration of lymphocytes and multinuclear leukocytes.

For reconfirmation of efficiency of essential oil on wound healing process, we investigated the microscopic structure of skin from burn wounds treated with essential oil, tamanu oil, and saline via histological examination (Figure 5). Histology studies revealed that burn wounds treated with essential oil developed complete epidermal structure: squamous epithelium covered on epidermis, the stratum spinosum keratinized, observation of matured hair follicles, thick and neatly arranged fibers, and scattered immune cells. That proved that burn wound treated with essential oil was fully recovered. In tamanu oil treated mice, wounds were partially recovered with existence of coagulative necrosis region on epidermis and vascular congestion, swollen stromal tissue, and no matured hair follicles. On the contrary, saline treated mice formed uneven epidermal layer with the necrotic ulcer on epidermis layer, infiltration of lymphocytes, plasmatocytes and multinuclear leukocytes, existence of granulation tissue, and fibrosis region. These results suggested that* C. operculatus *essential oil did not only accelerate the wound healing rate but also helped the wound recovery with normal structure.

## 4. Conclusion

We identified a total of 13 bioactive compounds of essential oil, several of which exhibited the anti-inflammatory and antimicrobial activities. Furthermore, the essential oil showed the antibacterial effect against* S. aureus*, the common pathogen bacterium in skin. The supportive effect of essential oil on burn wound healing process also has been proven. Of note, wounds of essential oil treated group were fully recovered. Furthermore, we found that wound contract rate of tamanu oil treated mice was higher than control mice (0.22 ± 0.03 and 0.31 ± 0.02 cm^2^, respectively) after 20 days. Histological studies revealed that burn wounds treated with essential oil formed a complete epidermal structure. On the contrary, tamanu oil and saline treated burn wounds were partially recovered. Therefore, these data prove that essential oil exerts the supportive effect for wound healing process not only in acceleration of wound contraction rate but also in recovery of normal epidermis and dermis structure. This finding demonstrates the utilization of* Cleistocalyx operculatus* leaf essential oil as promising topical agent. Furthermore, it also sheds light on the application of aromatherapy from by-product of tropical plants for treating the dermatological trauma.

## Figures and Tables

**Table 1:** Identification of some bioactive compounds in essential oil of *Cleistocalyx operculatus* leaves.

Number	RT (min)	Compound name	Formulas	M.W.	NIST ref.	Bioactivities
(1)	9.498	Camphenol, 6-	C_10_H_16_O	152	141039	

(2)	9.767	Isopinocarveol	C_10_H_16_O	152	292836	Antimicrobial, anti-inflammatory, antioxidant [3]

(3)	10.416	p-Cymen-8-ol	C_10_H_14_O	150	290794	

(4)	10.595	(−)-Myrtenol	C_10_H_16_O	152	334014	Anti-inflammatory, antinociceptive [4]

(5)	10.785	I-Verbenone	C_10_H_14_O	150	141212	Antibacterial, anti-inflammatory, anticonvulsive [5]

(6)	10.897	cis-Carveol	C_10_H_16_O	152	291523	

(7)	15.416	Ethaneperoxoic acid, 1-cyano-4,4-dimethyl-1-phenylpentyl ethaneperoxate	C_16_H_21_NO_3_	275	66383	

(8)	16.098	(+)-Carotol	C_15_H_26_O	222	42544	Antifungal [6]

(9)	16.154	Caryophyllene oxide	C_15_H_24_O	220	156329	Analgesic, anti-inflammatory [7], anticancer [8]

(10)	17.273	(−)-Globulol	C_15_H_26_O	222	109228	Antimicrobial [9]

(11)	17.418	2-(4a,8-Dimethyl-2,3,4,4a,5,6-hexahydronaphthalen-2-yl)propan-1-	C_15_H_24_O	220	189031	

(12)	17.418	6-Isopropenyl-4,8a-dimethyl-1,2,3,5,6,7,8,8a-octahydro-naphthalen-2-ol	C_15_H_24_O	220	189102	

(13)	19.331	Longipinocarvone	C_15_H_22_O	218	151871	

**Table 2:** Burn wound areas of experimental mice after 10 days and 20 days.

Day	Remaining burned skin area (cm^2^)		
	Saline	Tamanu oil	Essential oil
0	1.08 ± 0.10^a^	1.07 ± 0.06^a^	1.05 ± 0.06^a^
10	0.73 ± 0.04^a^	0.57 ± 0.04^b^	0.43 ± 0.03^c^
20	0.31 ± 0.02^a^	0.22 ± 0.03^b^	0.00 ± 0.00^c^

^a,b,c^Values with different letters within the rows are significantly different (*p* < 0.05).

## Data Availability

The dataset supporting the results of this article is included within the article and its supplementary files.

## Supplementary Materials

Supplementary Materials The raw data that demonstrated the antimicrobial activity of essential oil and the acceleration effect of essential oil on wound contraction rate were available in excel sheets in the Supplementary Materials.
